# Differential Gene Expression Patterns in Blood and Cerebrospinal Fluid of Multiple Sclerosis and Neuro-Behçet Disease

**DOI:** 10.3389/fgene.2021.638236

**Published:** 2021-02-26

**Authors:** Olfa Maghrebi, Mariem Hanachi, Khadija Bahrini, Mariem Kchaou, Cyrine Jeridi, Samir Belal, Samia Ben Sassi, Mohamed-Ridha Barbouche, Oussama Souiai, Meriam Belghith

**Affiliations:** ^1^Laboratory of Transmission, Control and Immunobiology of Infections, Institut Pasteur de Tunis, Tunis, Tunisia; ^2^Faculty of Medicine, Tunis El Manar University, Tunis, Tunisia; ^3^Tunis El Manar University, Tunis, Tunisia; ^4^Laboratory of Bioinformatics, Biomathematics and Biostatistics-LR16IPT09, Institut Pasteur de Tunis, Tunis, Tunisia; ^5^Faculty of Science of Bizerte, University of Carthage, Jarzouna, Tunisia; ^6^Charles Nicolle Hospital, Tunis, Tunisia; ^7^Department of Neurology, Mongi Ben Hmida National Institute of Neurology, Tunis, Tunisia

**Keywords:** multiple sclerosis, neuro-Behçet disease, multivariate analysis, cerebrospinal fluid, IL-17, CD73, A2A receptor

## Abstract

Inflammatory demyelinating disorders of the central nervous system are debilitating conditions of the young adult, here we focus on multiple sclerosis (MS) and neuro-Behçet disease (NBD). MS is an autoimmune disorder of the central nervous system. NBD, a neurological manifestation of an idiopathic chronic relapsing multisystem inflammatory disease, the behçet disease. The diagnosis of MS and NBD relies on clinical symptoms, magnetic resonance imaging and laboratory tests. At first onset, clinical and imaging similarities between the two disorders may occur, making differential diagnosis challenging and delaying appropriate management. Aiming to identify additional discriminating biomarker patterns, we measured and compared gene expression of a broad panel of selected genes in blood and cerebrospinal fluid (CSF) cells of patients suffering from NBD, MS and non inflammatory neurological disorders (NIND). To reach this aim, bivariate and multivariate analysis were applied. The Principal Analysis Component (PCA) highlighted distinct profiles between NBD, MS, and controls. Transcription factors foxp3 in the blood along with IL-4, IL-10, and IL-17 expressions were the parameters that are the main contributor to the segregation between MS and NBD clustering. Moreover, parameters related to cellular activation and inflammatory cytokines within the CSF clearly differentiate between the two inflammatory diseases and the controls. We proceeded to ROC analysis in order to identify the most distinctive parameters between both inflammatory neurological disorders. The latter analysis suggested that IL-17, CD73 in the blood as well as IL-1β and IL-10 in the CSF were the most discriminating parameters between MS and NBD. We conclude that combined multi-dimensional analysis in blood and CSF suggests distinct mechanisms governing the pathophysiology of these two neuro-inflammatory disorders.

## Introduction

Multiple sclerosis (MS) and neurological Behçet disease (NBD) are two inflammatory recurrent diseases affecting the central nervous system (CNS). MS is defined as an immune-mediated neuroinflammatory neurodegenerative disorder of the CNS, characterized by pleocytosis and T-cell infiltration in the cerebrospinal fluid (CSF) (Wu and Alvarez, 2011; Wootla et al., 2012; Eisele et al., 2014). NBD, meanwhile, is associated with an inflammatory cascade and neutrophils’ infiltration in the CSF (Al-Araji and Kidd, 2009; Kalra et al., 2014). These two disorders share the feature of presenting episodes of relapses and remissions (Caruso and Moretti, 2018). Both also showed the same encephalomyelopathy, disseminated in time and space (Sefidbakht et al., 2014).

Despite these similarities, these two pathologies exhibited different cranial MRI lesions. Indeed, NBD patients present lesions in the brainstem extending to the diencephalic structure and basal ganglia region, while MS patients present lesions mainly localized in periventricular areas (Akman-Demir et al., 2003; Brownlee et al., 2017). Serum and CSF analyses have also been reported to discriminate between these two pathologies. Indeed, intrathecal oligoclonal IgG bands (OCB) were more common in MS than in NBD (Saruhan-Direskeneli et al., 2013; Deisenhammer et al., 2019). Moreover, epidemiological estimation suggested that MS is more prevalent for women (2:1 to 3:1) (Stadelmann, 2011; Leray et al., 2016), in opposition to NBD that affects men more frequently (Akman-Demir et al., 1999; Ideguchi et al., 2010; Houman et al., 2013; Acar-Özen and Tuncer, 2021).

However, there are overlaps in the clinical feature characteristics of these two diseases, particularly at the early stages. Therefore, this may result in a misleading diagnosis on the basis of clinical and radiological criteria. Due to the crucial role of the immunological response in the pathophysiology of these two diseases affecting the CNS, a better characterization of cellular markers would be of considerable clinical relevance and may help guide clinicians in choosing the appropriate treatment.

So far, comparative immunological studies between NBD and the relapsing-remitting (RR) form of MS (RRMS) have shown commonalities and differences in profiles at the immunological level. In the context of the Th1 response pathway, the predominantly secreted cytokines are mainly IFN-γ, IL-2, TNF-α, and IL-12 (Raphael et al., 2015). A comparative study between these two disorders reported elevated levels of CXCL10 and TNF-α in the CSF of NBD patients compared to RRMS, while no difference in IL-12 secretion was observed (Saruhan-Direskeneli et al., 2003; Aldinucci et al., 2018). Our previous work demonstrated no significant difference in the expression of IFN-γ and T-bet in the CSF from these two diseases (Belghith et al., 2018). The Th17 profile, regulated by a combination of TGFβ and IL-6 levels, plays a crucial role in the pathogenesis of neurological disorders (Qin et al., 2009). Retinoic acid-related orphan receptor-γt (ROR-γt) is the specific transcription factor of this cell lineage (Tang et al., 2018). Studies on the Th17 axis showed no difference in IL-8 and IL-17 secretion between NBD and MS patients (Saruhan-Direskeneli et al., 2003). However, Aldinucci et al. (2018) found elevated levels of IL-6 and IL-8 in NBD as compared to MS. In our previous study, we found no difference in ROR-γt and IL-17 expressions in the CSF of the two groups of patients (Belghith et al., 2018).

Th2 and T regulatory cells’ implication in the CSF of neurological disorders have been poorly investigated. The cytokine signature produced by Th2 cells are IL-4, IL-5, IL-13, and GATA3, which is the major regulator of Th2 differentiation. Studies on Th2 populations in the CSF have shown comparable expressions of GATA-3 and IL-4 between NBD, MS, and the control group (Belghith et al., 2018). The immunomodulatory Treg cells are known to suppress inflammation and to induce tolerance in diseases affecting the CNS (Duffy et al., 2018; Okeke and Uzonna, 2019; Sambucci et al., 2019). The master marker of Treg cells is the transcription factor Foxp3, and the major immunosuppressive cytokine is IL-10. Our team has previously proposed IL-10 as a discriminative marker between these two disorders (Belghith et al., 2018). Regulation could also be assessed by the CD39/CD73 pathway to generate adenosine secretion which binds to the A2A receptor expressed on the effector T-cells membrane surface (Antonioli et al., 2013). Our previous studies have pointed to the elevated expression of CD39 in CSF samples of both NBD and MS, while the CD73 expression was lower in NBD patients (Bahrini et al., 2020).

The previous investigations of cellular markers associated with these two disorders were mainly focused on one or two features. In our study, we aimed to analyze simultaneously multiple parameters that would better distinguish the two CNS disorders at the first stage. The expression levels of both blood and CSF genes related to the Th1, Th2, Th17, and Treg populations have been compared, using multivariate and univariate statistical analysis. Our findings expand the current understanding of the immune process mediating the two neurological disorders (i.e., MS and NBD) and emphasize new potential cellular biomarkers that would enable a more accurate and specific diagnosis.

## Materials and Methods

### Patients

Patients with neurological diseases were enrolled from the Neurology department of Charles Nicolle hospital and the National Institute of neurology Mongi Ben Hamida (Tunis, Tunisia) from 2017 to 2020. For this study, all recruited patients were sampled during the first clinical episode of acute disease prior to any immunomodulatory treatment. After a period of clinical follow up to validate the final diagnosis, 66 subjects were included, and they were selected according to clinical examination, magnetic resonance imaging (MRI), and CSF laboratory data (IgG index and oligoclonal IgG band). A total of 21 MS patients had RR form and fulfilled the revised McDonald Criteria (Polman et al., 2011). All MS patients met the following inclusion criteria: age > 18 years old, had the RR form of the disease, underwent conventional MRI (3Tesla) with Gd-enhancement and lumbar puncture, and had positive oligoclonal bands restricted to CSF by isoelectric focusing and immune-blotting methods. They were examined and scored according to Kurtzke’s Expanded Disability Status Scale (EDSS) to assess the clinical severity of the disease. The NBD group included 22 patients with parenchymal involvement which fulfilled the International Study Group Criteria for Behçet’s disease (O’neill et al., 1994). Pediatric patients under the age of 18 and those with non-parenchymal NBD were excluded from the study. The Control group consisted of 23 subjects with non-inflammatory neurological diseases (NIND) with a persistent headache that required a lumbar puncture excluding any inflammatory or infectious etiology. This project was approved by the ethical committee of the Institute Pasteur of Tunis and all patients provided written informed consent before their inclusion in the study.

### RNA Extraction and Quantitative Real Time PCR

Peripheral blood mononuclear cells (PBMCs) were purified from blood patients by Ficoll technique and stored in Trizol reagent. CSF cells were also resuspended in an RLT buffer supplemented with 2% of beta mercapto-ethanol. Cells from blood and CSF were stored at −80°C for RNA extraction using an RNA isolation kit (Qiagen) under RNase-free conditions, according to the manufacturer’s protocol. The quality and quantity of RNA were assessed using agarose gel electrophoresis and NanoDrop analysis. RNase-free DNase I treated RNA (Qiagen) was reverse transcribed using High-capacity cDNA reverse transcription kit (Applied Biosystems). This reaction was performed with the following parameters: 1 μg RNA for 25°C for 10 min, 37°C for 2 h, and 85°C for 5 min. Quantitative real-time PCR was carried out using the Applied Biosystems ABI PRIZM 7500 Real-Time PCR System. The cycling protocol was set as follows: 15 s at 95°C and 1 min at 60°C for 40 cycles. Each 20 μl reaction contained 10 μL of 2× Sybr Green, 0.5 μL of each primer (0.25 μM final concentration each), and 5 μL of cDNA. Relative quantification of mRNA levels was performed using glyceraldehyde-3-phosphate dehydrogenase (GAPDH) as an endogenous reference. Samples were run in duplicate in three independent experiments. In this study, we used targeting genes encoding the Th1 (T-bet, IFN-γ), Th2 (GATA3, IL-4), Th17 (ROR-γt, IL-17), inflammation (TNF-α, IL6, and IL-1β), T reg (Foxp3, IL-10, TGF-β, Il-12αP35, and Ebi3), and the purinergic axis (CD39, CD73, and A2A). HPLC-purified oligonucleotides primer sequences are reported in Supplementary Data 1. Upon completion of each run, a melting curve analysis was performed to check the specificity of the primers. On some occasions, the PCR products were additionally analyzed by agarose gel electrophoresis.

### Statistical Analysis

Demographic characteristics of patients and controls were compared with contingency tables for categorical variables and non-parametric *T*-tests for continuous variables.

The statistical analysis was performed using R 3.12. Missing data were inferred using the missMDA package (Josse and Husson, 2016). The obtained dataset was subjected to data transformation (*Z*-score normalization and centering by mean) prior to principal component analysis (PCA) (Lê et al., 2008). Scaled and centered means of gene expression by disease were clustered in a heatmap (Gu et al., 2016; Kuleshov et al., 2016). The hierarchical clustering was achieved based on complete linkage and Euclidean distance measure. Wilcoxon-Mann-Whitney signed-rank and Kruskal–Wallis tests adjusted by a Bonferroni adjustment were respectively used to determine whether the difference between two groups and more than two groups is significant. Pairwise correlations matrices were calculated based on the Pearson Coefficient. To evaluate the specificity and the sensitivity of the analyzed parameters between disease categories, we used the pROC R package (Robin et al., 2011). The pre-processing step included the removal of correlated and near-zero variance predictors using the caret R package (Kuhn and Others., 2008). Then, a binomial generalized linear model (GLM) for each parameter was trained on 75% of the data and tested on 25%. The code used in the statistical analysis is available at this link: https://github.com/mariemh23/Differential-gene-expression-neuroinflammatory.

## Results

### Patient Characteristics

We compared PBMCs and CSF cells’ mRNA expression data of patients with the RR form of MS disease and NBD with the parenchymal syndrome and a control group including subjects with non-inflammatory neurological diseases (NIND).

Samples were obtained from patients who had not yet received any treatments during their first episode of clinical symptoms. The definitive diagnosis of either MS or neuro-Behçet disease was confirmed after 1 to 12 months of follow-up. The demographic and clinical characteristics of the subjects included in the study are reported in Table 1. No difference was observed regarding the mean age between the three groups (NBD = 42.19; RRMS = 36.30; NIND = 42.11; *p* = 0.4121). As expected, MS was more prevalent for women (17:4 vs 9:13 in NBD). RRMS patients showed an increased proportion of intrathecal immunoglobulin (Ig) synthesis (IgG Index = 0.97; *p* < 0.0001), and more frequently the presence of oligoclonal bands (OCBs) compared to NBD and NIND (RRMS = 95.2%; NBD = 13.6%; NIND = 0%; *p* < 0.0001). There was no significant difference in the blood-brain barrier (BBB) disruption among the three groups as demonstrated by the CSF to albumin ratio (Table 1).

**TABLE 1:** Demographic and clinical characteristics of patients.

	RRMS	NBD	NIND	*p* value
No. of patients	21	22	23	
Sex ratio (F/M)	17/4	9/13	20/3	0.0014
Mean age at onset	36.30	42.19	42.11	0.4121
(Min-Max)	(19–52)	(24–60)	(18–76)	
CSF				
Oligoclonal bands (±)	20/1	3/16	0/23	<0.0001
	95.2%	13.6%	0%	
IgG Index	0.97 ± 0.5	0.57 ± 0.38	0.49 ± 0.05	<0.0001
CSF IgG (mg/l)	57.05	32.95	22.78	0.0161
CSF/Serum Albumin ratio (×10^–3^)	4.766	5.186	4.761	0,51
Clinical features				
Patients in relapse	All	All	–	
Patients under therapy	None	None	–	
EDSS score	1.77		–	
(Min–Max)	(0–4)			

*NBD, neuro-Behçet disease; RRMS, relapsing remitting multiple sclerosis; NIND, non-inflammatory neurological disease; F, female; M, male; EDSS, the Expanded Disability Status Scale; CSF, cerebrospinal fluid.*

### Distinct Expression Profiles of Blood and CSF Immune Genes Between RRMS, NBD, and NIND

#### CSF Expression Profile Distinguished Between NIND and Neuro-Inflammatory Diseases

**FIGURE 1:**
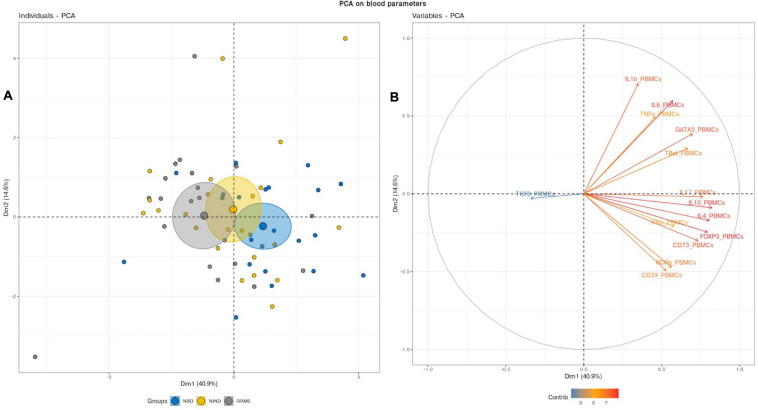
Blood variables fail to differentiate the NIND, NBD, and RRMS groups. **(A)** Individuals plot: samples are represented by dots and each color represents a group of patients. **(B)** Variables plot: vectors represent the 15 most influencing variables for each of the principal components PC1 and PC2. The longer the arrow is, the more it influences the variance. The ellipses indicate 95% confidence.

**FIGURE 2:**
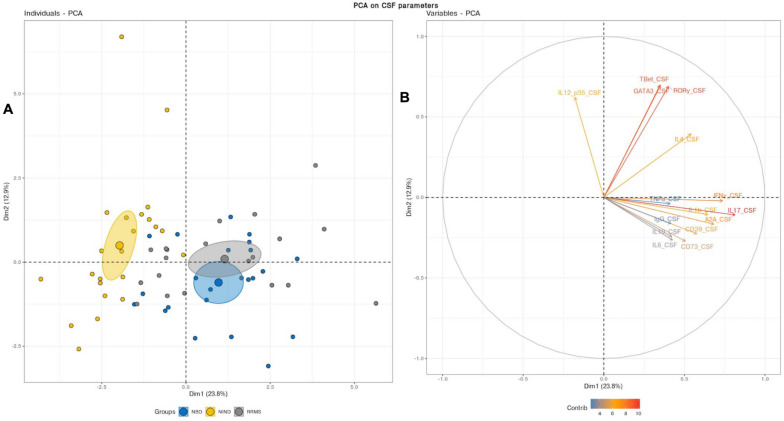
**(A)** Principal component analysis performed on CSF variables showed a distinct cluster between NIND and the neuro-inflammatory diseases. The samples are represented by dots. Each color represents a group of patients. **(B)** The vectors represent the 15 most influencing variables for each of the principal components PC1 and PC2. The longer the arrow is, the more it influences the variance. The ellipses indicate 95% confidence.

We first aimed to have an overview of the selected gene expression profile in patients diagnosed with RRMS and NBD as compared to the control NIND group by performing a PCA analysis. The individual plot of PBMCs RNA expression PCA did not segregate individuals into distinct groups, as illustrated by overlapping ellipses between RRMS, NBD, and NIND (Figure 1A). This observation suggests that PBMCs RNA expression variables alone fail to differentiate between the three groups (Figure 1B). In contrast, PCA of CSF parameters showed a statistically clear clustering between NIND and neuro-inflammatory diseases across the principal component PC1 (Figure 2A). This clustering was mainly attributed to IL-17, INF-γ, A2A, IL-1β, and CD39 expressions (Figure 2B). The above result might reflect an alteration of the expression of CSF related immune genes in the neuro-inflammatory diseases. It might be also explained by the infiltration of Th1 and Th17 effectors cells in the CSF compartment in inflammatory CNS diseases.

#### Blood Regulatory Marker Expression Is Distinct Between NBD and RRMS

**FIGURE 3:**
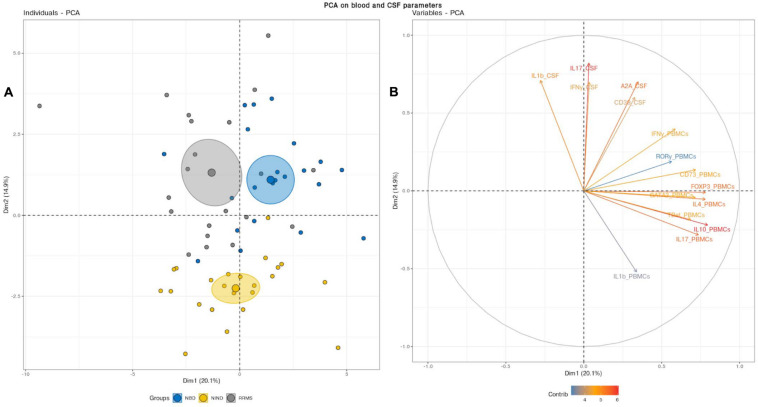
**(A)** Principal component analysis (PCA) performed on the full set of CSF and blood measured parameters significantly segregated the NIND, NBD, and RRMS groups. The samples are represented by dots. Each color represents a group of patients. **(B)** The vectors represent the 15 most influencing variables for each of the principal components PC1 and PC2. The longer the arrow is, the more it influences the variance. The ellipses indicate 95% confidence.

We deepened this analysis by mixing blood and CSF variables (Figure 3). This combination clearly discerned three clusters with non-overlapping confidence ellipses. Unsurprisingly, when analyzing the PCA, the CSF parameters segregated the inflammatory patients from the controls through the PC2 as reported above (Figures 2B, 3B). Most interestingly, the PBMCs RNA expression variables clearly drove the segregation between RRMS and NBD groups. Indeed, the main contributors to this clustering were genes encoding for regulatory T-cells, namely IL-10 and Foxp3, in addition to IL-4 and IL-17 (Figure 3B).

This latter observation suggests that NBD and MS do not show a significantly distinct CSF cell activation. Instead, NBD and MS exhibit a different expression pattern of blood parameters, more specifically, those related to the regulatory markers associated with the IL-17.

### Differential Immune Pathway Activation Trends Governing Physiopathology of RRMS and NBD Patients

#### Variation in Gene Expression Profiles in MS, NBD, and the Control Group

**FIGURE 4:**
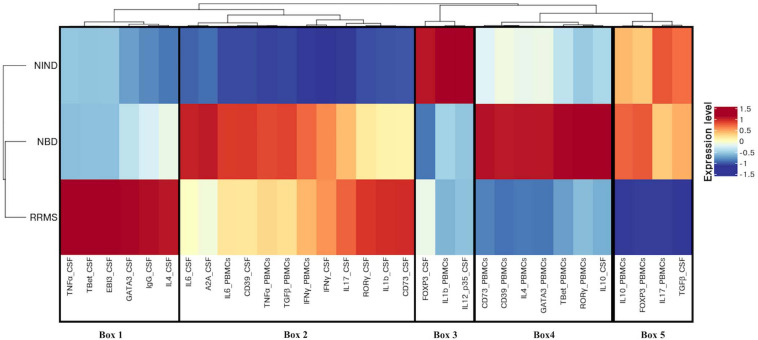
Different expression patterns of CSF and blood immune genes in NBD, RRMS, and NIND diseases. Feature expression heatmap was generated using the scaled and centered mean expression level of CSF and blood variables. Genes and disease groups were ordered based on hierarchical clustering. Boxes indicate the major distinct expression pattern and are detailed in the main text.

Then, we generated a feature-expression heatmap intending to identify specific differential expression patterns between the studied diseases and the NIND control group (Figure 4).

Following the hierarchical clustering of the gene expression pattern, the feature-expression heatmap was divided into 5 boxes, according to the most distinct expression patterns.

Compared to neuro-inflammatory groups, the NIND group exhibited a higher expression level of parameters linked to the regulation axis as Foxp3 and IL-12p35 in the CSF compartment (Box 3). The same expression trend was unexpectedly noticed for IL-1β in the blood (Box 3). In counterpart, genes related to inflammation, namely INFγ, TNF-α, and IL-6, were down-regulated in NIND PBMCs (Box 2). Moreover, gene expressions of variables associated with cellular activation (INFγ, IL-17, RORγ, GATA3, and IL-4) as well as variables associated with the purinergic system (A2A, CD73, and CD39) were down-regulated in NIND CSF (Box 2). This differential gene expression observed in the NIND group shows a decrease of gene expression linked to inflammation and lymphocyte differentiation, in line with an absence of inflammatory damage of the CNS in this control group.

As expected, RRMS patients showed increased expression of TNF-α, IL-1β, and intrathecal IgG synthesis in the CSF as compared to the other groups. Moreover, this group displayed activation of the Th1/Th2 and Th17 axes in the CSF. Interestingly, the RRMS expression profile showed a lower-expression of regulatory parameters (IL-10, Foxp3, and TGFβ) in the two compartments (Box 5).

In accordance with the systemic nature of Behçet disease, the NBD group demonstrated an exacerbated immune response in the blood. This is illustrated by an increased expression of inflammatory markers (IL-6 and TNF-α), effector related genes (GATA3, Tbet, and RORγt), and the regulatory parameters (Foxp3, CD39, CD73, and IL-10) (Box 4). In the CSF, we distinguished an up-regulation of well-characterized immune factors IL-6, IL-10, CD39, and A2A. However, Foxp3 represents the most down-regulated parameter in NBD CSF (Box 4 and 2).

#### Individual Expression Differences of Blood and CSF Parameters Among the Groups

To individually assess the statistical significance of the differentially expressed genes detected, we used the Wilcoxon and Kruskal–Wallis statistical tests (Supplementary Figures S1, S2 and Supplementary Table S1). We, therefore, confirmed the over-expression status of TNF-α in the CSF of RRMS patients compared, respectively, to NBD (*p* = 0.0034) and NIND (*p* = 0.0049) (Supplementary Figure S1). Similarly, the comparison between RRMS, NIND, and NBD groups in the CSF showed significant over-expression of IL-10 (NBD vs RRMS: *p* < 0.0001; NBD vs NIND: *p* = 0.0011), CD39 (NBD vs RRMS: *p* = 0.031; NBD vs NIND: *p* < 0.0001), and A2A (NBD vs RRMS: *p* < 0.0001 NBD vs NIND: *p* < 0.0001) (Supplementary Figure S1). Furthermore, we noted an elevated expression of CD39 and CD73 in PBMCs of NBD patients (Supplementary Figure S2).

#### Correlations Analysis Within-Groups Reveal Shared and Unique Patterns

**FIGURE 5:**
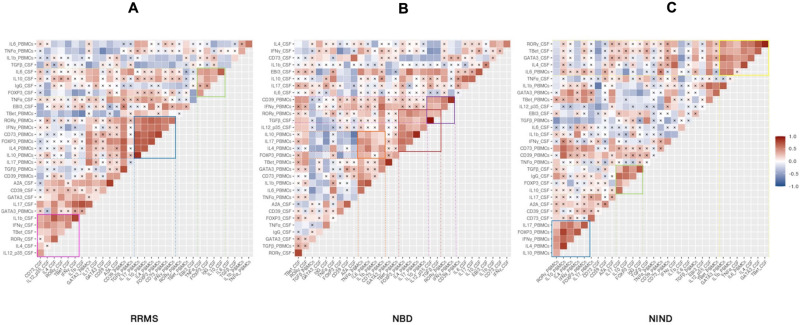
Specific CSF and blood genes co-regulation implicated in physiopathology of inflammatory and non-inflammatory diseases. The heatmap illustrates Pearson’s correlation between gene variables expressed in RRMS **(A)**, NBD **(B)**, and NIND **(C)** disease. Genes were ordered based on hierarchical clustering. The color intensity is proportional to the magnitude of the correlation coefficient. Positive correlations are displayed in red, negative correlations are colored in blue. Crosses indicate a non-significant correlation (*p* < 0.05).

Having described the differential expression levels, we next investigated the interdependence of the studied genes (Figure 5). We found that, among the whole set of parameters, a small proportion of studied genes was significantly correlated (Figure 5). We noticed the presence of gene clusters that are shared by disease groups. Among these clusters, we observed a module including common co-expressed gene patterns between NIND and RRMS patient groups. These genes were functionally related to Th1 and Th17 (IFN-γ and IL-17) and regulation (IL-4, IL-10, and Foxp3) in the blood (Figures 5A,C). Except for Foxp3, the latter module was also observed in the NBD group and additionally included the CSF parameters IL-12p35 and TGFβ (Figure 5B).

Another significant module was visualized in both CSF correlograms of RRMS and NIND groups. This module involved regulatory markers forming a positive correlation with inflammation markers IL-6 and IL-17 in RRMS and NIND groups, respectively (Figures 5A,C). Interestingly, in the NBD correlogram, we noticed that IL-10 was negatively correlated with Foxp3, CD39, and A2A (Figure 5B and Supplementary Figure S1).

Otherwise, the correlograms emphasized specific correlation modules for either NIND or RRMS. More precisely, we observed a positive correlation between genes related to Th1, Th17, and IL-1β immune axis in CSF of RRMS patients (Figure 5A). Regarding the NIND group, CSF and PBMCs markers associated with inflammation and cell activation formed a cluster of correlation (Figure 5C). The latter observation is almost certainly due to the down-regulation of those parameters in both compartments (Figure 4).

### Specific Blood Parameters as a Potential Discriminatory Marker for RRMS and NBD

We used the area under the curve (AUC) of ROC analysis aiming to evaluate the discriminatory strength of CSF and blood parameters between the groups of diseases. Indeed, the AUC in a ROC analysis can be used to measure the quality of a diagnostic test ranging from acceptable (AUC 0.7–0.8) through to excellent (AUC 0.8–0.9) and outstanding (AUC > 0.9).

**FIGURE 6:**
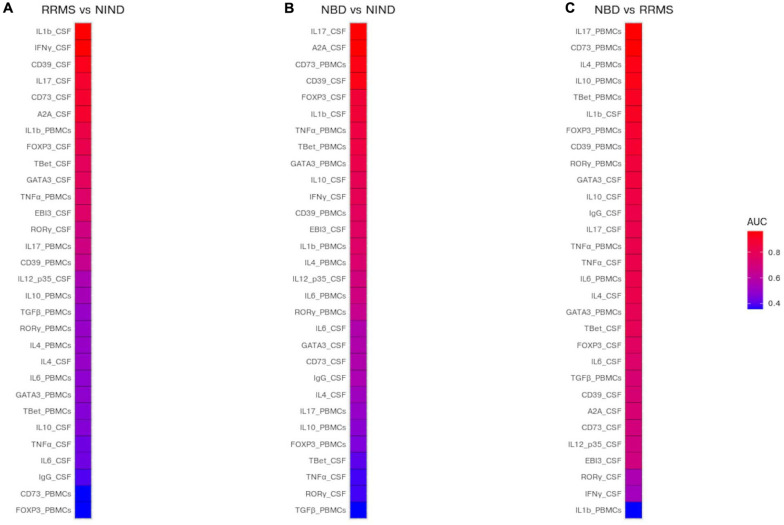
Prediction of potential biomarkers to discriminate between NBD, RRMS, and NIND. The AUC was estimated for each CSF and blood variable and visualized in a heatmap. Color reflects the discriminatory strength of the variable between RRMS vs NIND **(A)**, NBD vs NIND **(B)**, and RRMS vs NBD **(C)**. An AUC of 1 means that the variable perfectly discriminates between patients, whereas an AUC of 0.5 means that the variable is uninformative.

The performance of each predictor in discriminating each pair of disease groups was assessed by measuring the AUC (Figure 6 and Supplementary Table S2). We first estimated the investigated genes to differentiate between each group of patients from the control group. The most discriminatory and outstanding predictors were exclusively expressed in the CSF, namely IL-1β, INFγ (AUC = 0.96), and the CD39 (AUC = 0.92) for NIND and RRMS (Figure 6A). Regarding the comparison between NIND and NBD, CSF IL-17 (AUC = 0.97) and A2A (AUC = 0.97), followed by CD39 (AUC = 0.94) and the blood parameter CD73 (AUC = 0.94) showed a strong predictive power (Figure 6B).

The most relevant finding was the outperformance of blood parameters compared to those expressed in CSF to discriminate between NBD and RRMS patients (Figure 6C). These parameters are IL-17 (AUC = 0.96), CD73 (AUC = 0.96), IL-4 (AUC = 0.93), and IL-10 (AUC = 0.93) (Figure 6C).

## Discussion

The overlapping symptoms and MRI findings between MS and NBD renders diagnosis difficult at the early stage of the disease. Here, we investigated the expression genes related to the major immune axes in blood and CSF compartments of treatment-naive patients suffering from RRMS and NBD. We first performed a multivariate analysis using combined blood and CSF parameters; we observed three distinct clusters corresponding to the three studied groups.

These clusters are not identified when blood PCA analysis alone is performed. Interestingly, individual CSF PCA analysis distinguishes patients from both RRMS and NBD from the NIND control group, but not from each other. Our results highlight the preponderant role, at the expression level, of IL-17 and regulatory markers IL-4, IL-10, and Foxp3 in the blood as the major contributors to the segregation of the two studied neurological disorders. In addition, by further exploring the physiopathological signaling events, we observed a fine-tuning of T-cell activation localized in the CSF of RRMS patients, in contrast to a more generalized inflammatory response in the blood and CSF of NBD patients. Finally, comparisons with the control group showed that RRMS and NBD patients are characterized by the activation of Th1 and Th17 effectors cells and the elevation of A2A receptor in the CSF compartment.

In line with previous reports, NBD patients present an enhanced expression of T-bet (Th1) and RoR-γt (Th17) and a low expression of GATA 3 (Th2) (Hamzaoui et al., 2011; Mnasria et al., 2020). The secretion of these two inflammatory cytokines, IFN-γ and IL-17, in tissue-specific lesions was reported as implicated in the persistence and the evolution of Behçet disease (Zhou et al., 2012). On the other hand, MS disorder is believed to be induced by the activation of infiltrating lymphocytes, major producers of IFN-γ, within lesions. The over-expression of IFN-γ, particularly during acute exacerbation phases, has apoptotic effects on human oligodendrocytes. Moreover, the INF-γ signaling in astrocytes triggers the expression of a wide range of chemokines involved in the recruitment of inflammatory cells to the CNS (Pouly et al., 2000; Huang et al., 2004). While the Th1 causes primary spinal cord inflammation, the Th17 was described as an efficient inducer of predominantly brain damage (Stromnes et al., 2008). Indeed, Th17 cells accumulation was previously reported in active lesions of the CNS, while increased IL-17 mRNA and protein levels were observed in lymphocytes, astrocytes, and oligodendrocytes (Tzartos et al., 2008; Wang et al., 2011).

More interestingly, the enhanced expression of A2A observed in the CSF of the two neuro-inflammatory diseases is consistent with previous reports that described the up-regulation of A2A on T cells, macrophages, and microglia within the inflamed CNS tissue (Rebola et al., 2011). In addition, studies using A2A receptor antagonists in an experimental autoimmune encephalomyelitis model demonstrated the key role of this receptor in controlling different neurological diseases and in the prevention of neuroinflammation (Ingwersen et al., 2016).

The pathogenesis of NBD is poorly understood and the differentiation from other neuro-inflammatory diseases based on clinical and cellular markers remains challenging at the early stage of the disease. We herein identified, for the first time, the best predictors of NBD in both PBMCs and CSF compartments. Specific gene expression indicates elevated IL-6, IL-10, CD39, and A2A in the CSF and an exacerbated immune response in the blood of NBD compared to MS and NIND. These results are in accordance with data previously reported showing increased T cell activation and inflammation in the blood of patients suffering from various manifestations of Behçet disease, expressing higher levels of IL-1β in synovial fluid compared to that in osteoarthritis patients (Pay et al., 2006; Zhou et al., 2012). Furthermore, IL-6 has been reported to be associated with the disease activity and markedly elevated in the CSF of progressive NBD (Hirohata et al., 1997; Aridogan et al., 2003; Akman-Demir et al., 2008; Saruhan-Direskeneli and Direskeneli, 2021). We also identified differences in gene expression profiles of MS disease as compared to NBD. The most notable finding is the activation of Th1/Th2 and Th17 cells in addition to TNF-α and IL-1β expressions in the CSF of RRMS. This observed change in CSF cell gene expression is in accordance with previously described reports where MS patients had altered cellular composition of their CSF as compared to non-inflammatory neurological diseases. Interestingly, up-regulated genes are mostly observed in the CSF compartment while profiling in peripheral blood cells may not be as relevant. Indeed, the observation of the scarcity of differentially expressed transcripts in PBMCs from MS patients compared to controls was already described (Cepok et al., 2001).

The most differentially expressed transcripts in the CSF between MS and controls are, as expected, transcripts related to immunoglobulins as previously suggested (Achiron and Gurevich, 2006; Brynedal et al., 2010). The CSF profile of MS exhibits high levels of molecules linked to B cells’ recruitment and maturation as well as mediators related to B cells’ immune functions and molecules involved in lymphoid neogenesis and apoptosis (Brynedal et al., 2010; Magliozzi et al., 2018). Furthermore, studies using large-scale sequencing of cDNA libraries derived from brain plaques dissected from patients with MS identified an abundance of transcripts for osteopontin (OPN), a regulator of Th1 cells, and would be a promising therapeutic target to decelerate the development of progressive MS (Chabas et al., 2001).

The AUC of ROC analysis identified IL-17 and CD73 expressions in PBMCs as the strongest discriminant parameters, distinguishing RRMS from NBD with high accuracy. In the blood of Behçet disease patients, an elevated level of IL-17 was described more frequently in those developing neurological involvement and is considered as a marker of disease progression (Al-Zifzaf et al., 2015; Belghith et al., 2018). However, in MS, IL-17 was predominantly found in the CSF and *in situ* lesions (Matusevicius et al., 1999; Kebir et al., 2007; Tzartos et al., 2008). We speculated that this decreased expression of IL-17 in the blood of MS patients is probably due to the different compartmentalized inflammation processes, where Th17 cells are lower in the blood of MS patients since they migrate within the CNS. It has been demonstrated that the first wave of infiltrating cells in MS was Th17 cells (Korn et al., 2007) whilst neutrophil infiltration governs the pathophysiology of NBD (Arai et al., 2006).

In this study, we also focused on the purigenic mechanism which implicates the two ecto-enzymes CD39 and CD73. In the blood compartiment, we showed that, although up-regulated in NBD, the CD73 was significantly decreased in MS patients and correlates with Foxp3 (Bahrini et al., 2020). In line with observations in NBD, a subpopulation of peripheral CD73+CD4+ T cells enriched with IL-17 cells during active inflammation was described in inflammatory bowel disease (Doherty et al., 2012).

Interestingly, the most discriminatory and outstanding CSF predictor between these two diseases is the IL-1β. This potentially argues in favor of the infiltration of inflammatory monocytes into the CNS of MS patients, since IL-1β promotes their differentiation into antigen-presenting cells to activate CD4 reactive cells (Paré et al., 2018). In agreement with these observations, we noted a positive correlation between IL-1β and effector T-cells related genes in the CSF RRMS patients. Furthermore, lower levels of IL-1β gene expression in the CSF of NBD patients is in agreement with data showing that IL-1β and TNF-α were not elevated in the CSF of patients with an acute or chronic progressive form of NBD (Hirohata and Kikuchi, 2012).

The multivariate approach on a set of key genes, used in the current study, allowed for the most comprehensive insights into expression profiles in CSF and blood of MS, NBD, and NIND. During the acute phase of the first onset of the diseases, the CSF of these two neuro-immunological disorders shows an activation of Th1, Th17, and the purinergic axes CD39/CD73. Interestingly, our model predicted blood IL-17 and CD73 expression as discriminative markers between those two pathologies.

Nevertheless, we admit that this study is subject to certain limitations; indeed, the number of studied parameters could be further extended to better assess the potential role of other diagnostic markers for differentiating MS and NBD. We also acknowledge that a higher number of patients would increase the statistical power of this study. Our priority was to include patients based on strict selection criteria used during patients’ recruitment.

In conclusion, the most striking finding in this study is the identification of genes that contribute to the pathophysiology of MS and NBD. We differentially characterize patients with NBD from those with MS and the controls. These clusters combined both genes expressed in blood and CSF. These data could pave the way to validation of specific mRNA signatures which could help establish differential diagnosis at early stages of the disease.

## Data Availability Statement

The original contributions presented in the study are included in the article/Supplementary Material, further inquiries can be directed to the corresponding author/s.

## Ethics Statement

The studies involving human participants were reviewed and approved by Ethics Committee of Pasteur Institut of Tunisia. The patients/participants provided their written informed consent to participate in this study.

## Author Contributions

All authors listed have made a substantial, direct and intellectual contribution to the work, and approved it for publication.

## Conflict of Interest

The reviewer KH and the authors OM, KB, SB, SBS, M-RB, OS, and MB are not members of the same institution, but both their institutions are affiliated to the same university. The reviewer KH declared this shared affiliation with authors OM, KB, SB, SBS, M-RB, OS, and MB to the handling editor at time of review. The remaining authors declare that the research was conducted in the absence of any commercial or financial relationships that could be construed as a potential conflict of interest.

## Supplementary Material

Supplementary Figure 1 Boxplots representation of CSF parameters expression per disease.

Supplementary Figure 2 Boxplots representation of blood parameters expression per disease.
