# Supplementary figures and images for: Differential Gene Expression Patterns in Blood and Cerebrospinal Fluid of Multiple Sclerosis and Neuro-Behçet Disease
Source: Front Genet. 2021 Feb 26;12:638236. doi: 10.3389/fgene.2021.638236 (PMC7954360; doi:10.3389/fgene.2021.638236)

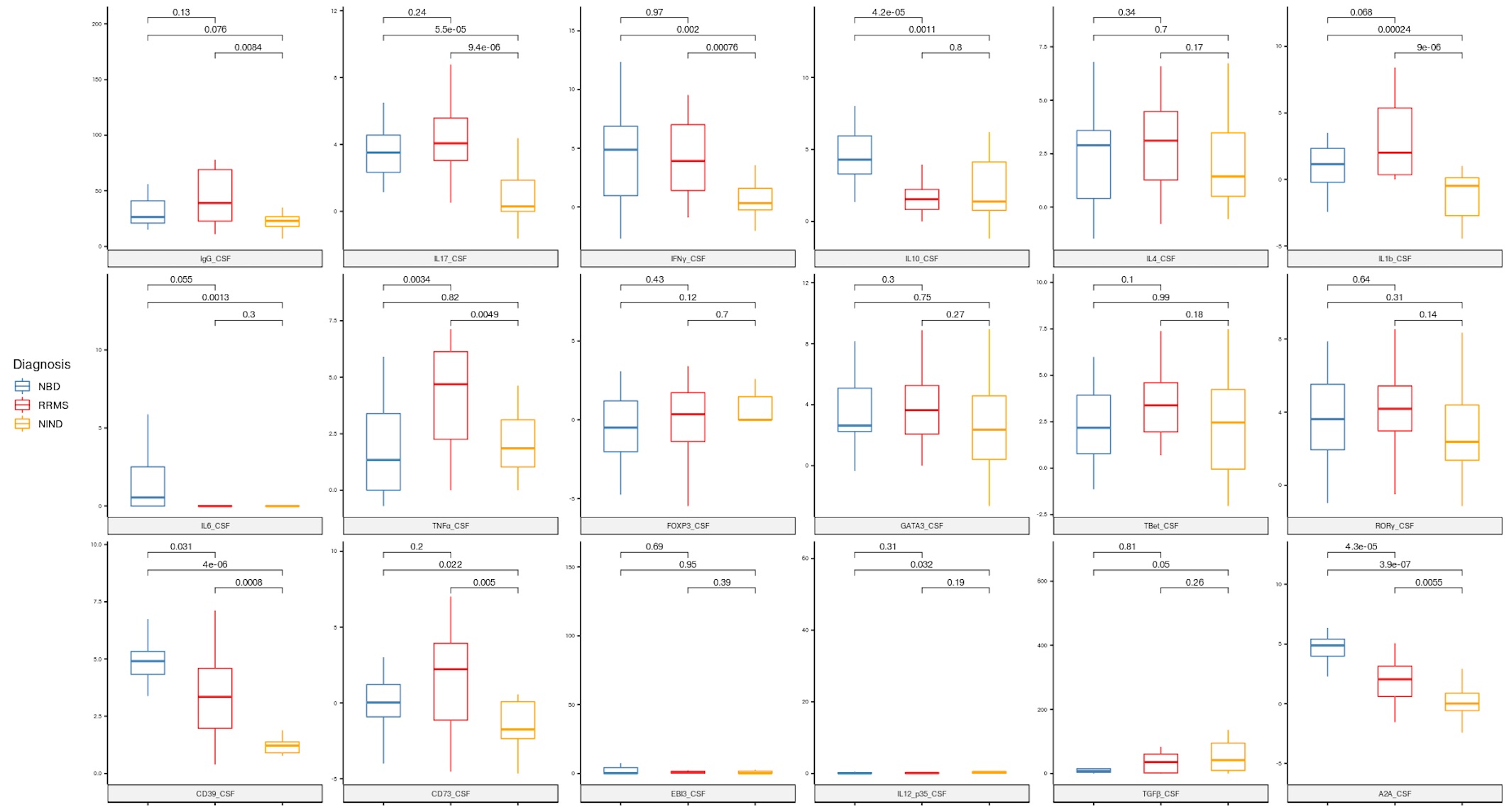

Supplement: Supplementary Figure 1 — Boxplots representation of CSF parameters expression per disease. [file Image_1.JPEG]

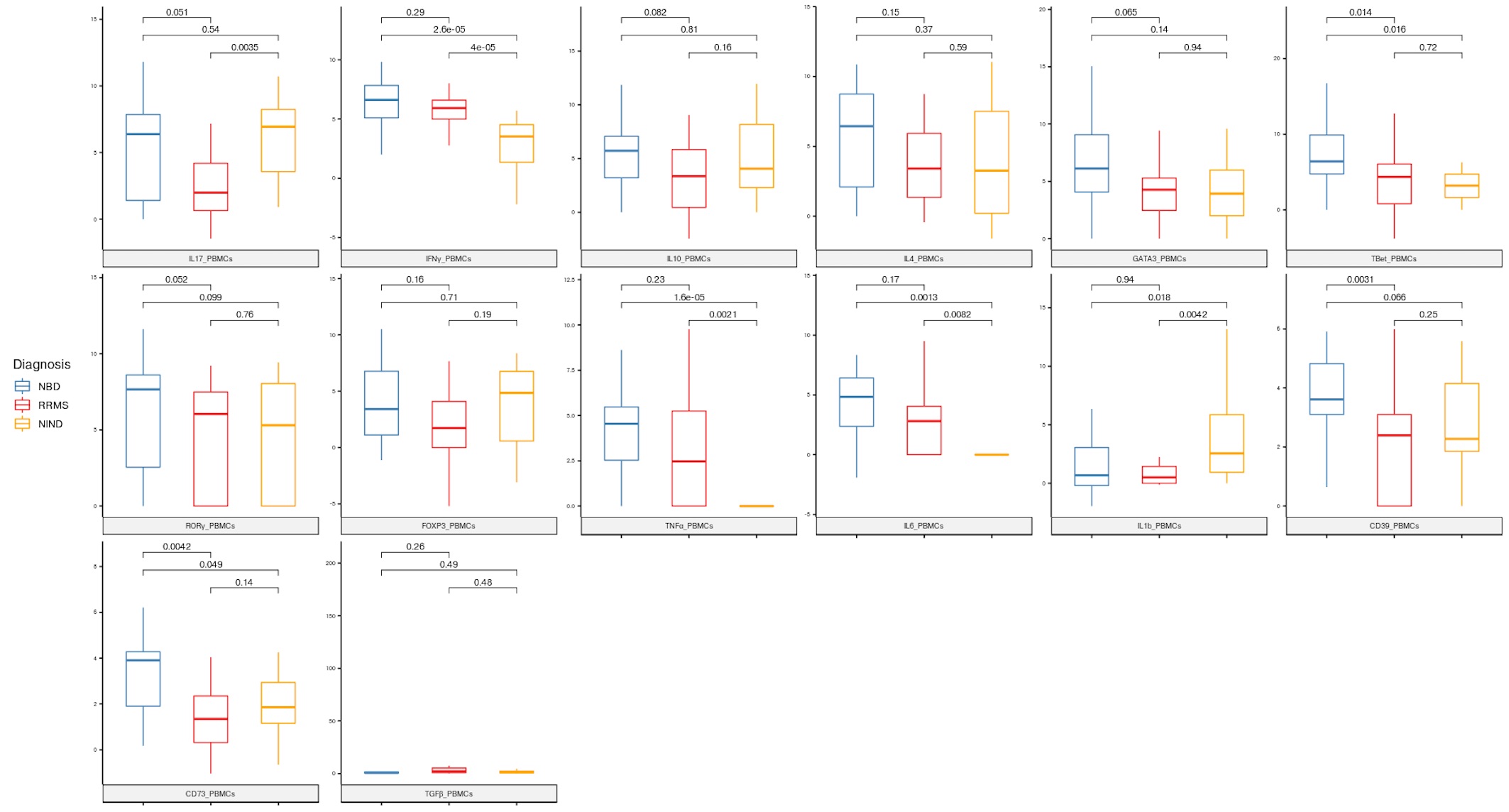

Supplement: Supplementary Figure 2 — Boxplots representation of blood parameters expression per disease. [file Image_2.JPEG]
