# Supplementary material for: Differential Gene Expression Patterns in Blood and Cerebrospinal Fluid of Multiple Sclerosis and Neuro-Behçet Disease
Source: Front Genet. 2021 Feb 26;12:638236. doi: 10.3389/fgene.2021.638236 (PMC7954360; doi:10.3389/fgene.2021.638236)
Supplement: Supplementary file 5 [file Table_3.DOCX]

| **Gene** | **Forward** | **Reverse** |
| --- | --- | --- |
| **Foxp3** | 5’-AGCTGGAGTTCCGCAAGAAAC-3’ | 5’-TGTTCGTCCATCCTCCTTTCC-3’ |
| **IL-4** | 5’-CACGGACACAAGTGCGATA-3’ | 5’-GATGTCTGTTACGGTCAACTCG-3’ |
| **IL-10** | 5’-CGAGATGCCTTCAGCAGAGT-3 | 5’-CCCTTAAAGTCCTCCAGCAA-3’ |
| **IFN-γ** | 5’-TTTGGGTTCTCTTGGCTGTT-3’ | 5’-TCCATTATCCGCTACATCTGAA-3’ |
| **IL-17** | 5’-ACCAATCCCAAAAGGTCCTC-3’ | 5’-TGGATGGGGACAGAGTTCAT-3’ |
| **T-bet** | 5’-AGGATTCCGGGAGAACTTTGAG-3’ | 5’-AATTGACAGTTGGGTCCAGGC-3’ |
| **GATA3** | 5’-CAAAATGAACGGACAGAACCG-3’ | 5’-GCTCTCCTGGCTGCAGACA-3’ |
| **ROR-γt** | 5’-AATGACCAGATTGTGCTTCTCAAAG-3’ | 5’-GGTTGTCAGCATTGTAGGCCC-3’ |
| **CD39** | 5’-CTGATTCCTGGGAGCACAT-3’ | 5’-GACATAGGTGGAGTGGGAGAG-3’ |
| **CD73** | 5’-CGCAACAATGGCACAATTAC-3’ | 5’-CTCGACACTTGGTGCAAAGA-3’ |
| **TNF-α** | 5’-CAGAGGGAAGAGTTCCCCAG-3’ | 5’-CCTTGGTCTGGTAGGAGACG-3’ |
| **IL6** | 5’-ATGAACTCCTTCTCCACAAGCGC-3’ | 5’-GAAGAGCCCTCAGGCTGGACTG-3’ |
| **Il-12αP35** | *5′-CCACTCCAGACCCAGGAATGT-3′* | 5′-CCTCCACTGTGCTGGTTTTATCT-3′ |
| **Ebi3** | 5′-TCATTGCCACGTACAGGCTC-3′ | 5′-GGGTCGGGCTTGATGATGTG-3′ |
| **A2A** | 5′-AGTTCCGCCAGACCTTCC-3’ | 5′-ACCTGCTCTCCGTCACTG-3’ |
| **TGFB** | 5′-GCCCTGGACACCAACTATTG-3’ | 5’-CTGGTCCAGGCTCCAAAT-3’ |
| **IL-1β** | 5’-AGGGACAGGATATGGAGCAACAAG-3’ | 5’-CATCTTTCAACACGCAGGACA-3’ |
| **GAPDH** | 5’-CCACATCGCTCAGACACCAT-3’ | 5’-GGCAACAATATCCACTTTACCAGAGT-3’ |
